# The Foreign Journals: Pathology, Practical Medicine, and Therapeutics

**Published:** 1838-10

**Authors:** 


### PATHOLOGY, PRACTICAL MEDICINE, AND THERAPEUTICS.

### *Cauterization of the Pharynx in Croup.* By Dr. Felix Hatin.

It appears this remedy was first tried by Dr. Peronneau in the treatment of laryngeal and pharyngeal inflammations, and Dr. Hatin has since used it in four cases of incipient croup [?] with complete success. The first case given is that of a child five years of age, who was seized with that peculiar hoarse cough so difficult to compare, but so easily recognized when once heard, and which indicates the commencement of croup. Leeches were immediately applied; and on fears being expressed to the father regarding the termination, he related the case of a child who had been cured of an attack of croup by cauterization practised by M. Peronneau. M. P. was then called to the present case, and the following course was adopted. The child was placed upon the father's knees, who with one hand fixed the arms, and with the other retained the head against his chest. The operator placed himself in front, holding in his left hand an instrument necessary to keep the mouth open and to depress the tongue, and in the right a *porte-pierre,* bent like a sound and containing a piece of nitrate of silver, projecting some lines. The tongue being depressed, the tube containing the nitrate of silver was passed into the posterior fauces and rapidly passed over all points for a second or two; the two instruments were then withdrawn to allow of respiration. Some minutes after, a second cauterization, similar to the first, finished the operation. The child did not complain of much pain or uneasiness. The next morning, after a quiet night, the cough was simply catarrhal, and the patient out of danger. Upon looking into the throat, the tonsils, soft palate, posterior wall of the pharynx, and all the points accessible to the sight were covered with a white eschar, which after a few days disappeared, leaving a bright red appearance, not causing sufficient pain to embarrass deglutition.

Another case is related, occurring in the son of M. Imard director of the Hospital "La Pitié." This child, nine years and a half old, was seized in the night with the premonitory symptoms of croup, and Dr. Hatin was summoned in the morning. He at once determined to practise cauterization, but first requested the advice of M. Serres, whose opinion was similar to his own. The application was made in exactly the same way as the last case. In the evening the croupy cough had disappeared, and that which remained gradually diminished in intensity.

Two other cases are given in which this method of treatment was equally efficacious. To ensure success, it is necessary that the cauterization should be performed during the first few hours of the attack, for if the false membrane occupy the larynx or trachea the application will be ineffectual, at least Dr. H. has employed it in two such cases, and it exerted no influence on the progress of the disease.

*Revue Médicale. October,* 1837.

---

### *On the Employment of the Hydrochlorate of Tin.* By Dr. Schlessinger.

Dr. S. recommends this medicine as exerting a peculiar soothing power over the nervous system, and also as a remedy in some diseases of the skin. The dose is from a sixth to a quarter of a grain three or four times a day; or a grain may be dissolved in one drachm of muriatic ether, and five drops taken three or four times a day, and increased daily, till the dose is two grains. Sometimes it increases the symptoms it is given to combat; however, this may be considered as a favorable omen. When the patient complains of dryness of the throat, fever, and gastro-intestinal irritation, the remedy should be suspended, or at least the dose diminished. The first case reported, is that of a young man twenty-two years of age, who, for ten months had been subject to epilepsy. The hydrochlorate of tin dissolved in muriatic ether was given, but also a decoction of bark. The convulsions became stronger, and the skin covered with a miliary eruption. The medicine was continued, and the patient cured in about five weeks. Another case is related of an eruption upon the face and hands, which resisted all the means used. After taking the medicine for three weeks, the symptoms increased, but two weeks after they disappeared, and the disease was completely cured.

*Journal des Connaisances Médico-Chirurgicales. Avril,* 1838.

---

*On Epidemic Epilepsy in Schools.* By Dr. MEYER.

THE free school of Bielefeld is a well-aired, not overcrowded room, in which the boys and girls are taught at the same time. A young girl of the name of Arnold had for some time been subject to epileptic fits, and had been repeatedly seized during the school hours, on which account she was forbidden to attend. Apparently restored to health she was again admitted, but on the 8th of August, 1837, she was again seized, and was in consequence carried home. A few days afterwards a strong healthy girl who had occasionally accompanied Arnold home was seized with convulsions in the school-room; on the 14th two other girls, aged respectively twelve and fourteen years, were affected in a like manner; but this did not prevent them from making their appearance at school on the following morning. Scarcely, however, had the business of the day commenced, when not only these two, but likewise three other girls, were affected with epileptic convulsions, and the contagion spread with such rapidity that in less than half an hour above twenty girls were similarly affected.

At first the children experienced a feeling of anxiety; they were then observed to grow pale, there was oppression of the chest, and the head became affected; trembling of the limbs followed with loss of consciousness; the thumbs were bent upon the palms, the eyes were distorted, and the patient gave vent to a sudden anxious cry. The paroxysm in some was of short duration, but in others it continued for hours. None of the boys were attacked. The temperature of the room was about 18° R. (72° F.) at noon. None of the girls attacked, except Arnold, had ever previously had an epileptic paroxysm, and no material cause for the disease could be discovered. Most of the girls were approaching the age of puberty, and they were all of a highly excitable temperament.

Notwithstanding that the girls who had been affected were not allowed to return to the school for a considerable time, new cases afterwards occurred, owing, it was suspected, to some of the girls who were not completely reestablished having been readmitted and having suffered from fresh paroxysms. The disease was treated as purely nervous, with valerian, oxide of zinc, indigo, &c.; but on the whole with little success, for after the lapse of five months there were very few who could be considered as safe against a relapse.

The number of sufferers in the school of Rietberg was not so numerous, but was sufficient to show the facility with which convulsive diseases may be communicated to feeble and excitable constitutions.

A gir., aged twelve years and a half, had during two previous years suffered occasionally from epileptic paroxysms. Towards the end of May, 1837, she had an attack in the school, and shortly afterwards four others were similarly affected. In these cases no premonitory symptoms were observed; the patients uttered a shriek and fell insensible to the ground, convulsions generally followed, sometimes alternating with tonic spasms, but in the four last cases there was no foaming at the mouth, nor were the thumbs contracted. The paroxysms lasted about a quarter of an hour, rarely half an hour. The disease appeared to be little under the influence of medical treatment, at least the remedies employed did not seem to have any beneficial effect. *Medicinische Zeitung.* No. 8. 1838.

*Treatment of Spermatorrhœa.*

CASE I. A young man, æt. twenty-three, suffered both bodily and mental debility from involuntary discharge of semen, every night. Antiphlogistic remedies, and subsequently tonics, were employed without any benefit. The extract of lettuce, prepared in the manner recommended by M. Caventon was given, in doses of from two to eight grains daily. The amelioration was rapid, and the patient was completely cured in a fortnight.

CASE II. A similar case was treated with like success with camphor, in the following formula:—Twenty grains of camphor dissolved in almond oil, and made into twenty pills, by means of gum arabic. One was taken every night, and every fourth day the dose was increased by one pill, until four pills were taken daily. The spermatorrhœa, which had existed during three years, and which had resisted various medicines, completely ceased in eight days after taking the above pills, which were continued eight days more, and the cure was complete.

*Gazette des Hopitaux.* No. 52. 1838.

---

*On Artificial Auscultation.* By M. PETREQUIN.

THE difficulty attending the commencement of the study of auscultation, and the great number of observations required before the student is enabled to appreciate justly the great variety of sounds which may be recognized in the diseased lung, have induced M. Petrequin, surgeon of the Hôtel Dieu at Lyons, to invent a method of artificial auscultation. By a simple contrivance he is enabled to reproduce after death the sounds which are characteristic of the diseases which have proved fatal, and thus to afford a great facility to the student. In his first experiments he removed the lungs from the body, and by applying a pair of bellows to the trachea, he produced in the healthy lung a vesicular murmur precisely similar to that heard during life. Afterwards, by injecting liquids of different densities and then inflating as before, he was enabled to produce the several mucous râles or rhonchi. In the case of a man who had died of pneumonia, he inflated the lungs after removing them, and produced crepitous râle with bronchial respiration. He next commenced artificial auscultation of the lungs without removing them from the body. For this purpose he opened the larynx or trachea, and inserting the pipe of the bellows, he carefully closed the opening. He found it convenient to make use of a bellows with a syringe attached, as the sounds became occasionally obscured by mucosities drawn from the minuter air cells, and the syringe served to remove these. M. Petrequin now began to auscultate the bodies of those with whose symptoms he had not been acquainted during life, and he constantly found his diagnosis verified by the inspection which was afterwards made. Thus in cases of pneumonia he found absence of respiratory murmur, and crepitating râle more or less intense according to the degree and period of the inflammation. In hydrothorax he found the pulmonary sounds obscured by the fluid in the chest, and also modified by the position of the body. In phthisis he found dullness, bronchial respiration, and mucous rhonchus or *gargouillement.* Encouraged by this success, he endeavoured to produce an artificial voice in the corpse; but his first efforts were not successful. He introduced a tube into the larynx, and afterwards a metallic funnel into the trachea; but on speaking through them he found that there was no resonance in the chest. At last he succeeded by placing one end of the stethoscope on the larynx of a person speaking in a loud voice, the other end being applied to the division of the bronchi in the subject, and artificial respiration being kept up. This artifice produced resonance of the voice as well as of cough in the chest.

M. Petrequin has found artificial auscultation of the greatest use to himself in the study of the pulmonary sounds. Here (he says) is no patient to be fatigued and have his symptoms aggravated by a long and careful examination; here all distracting noises may be removed, and the experimenter will have the great advantage of being able immediately to verify or to correct his diagnosis, and to assign the physical causes to the respective sounds with a degree of certainty which cannot be obtained during life on account of the organic changes produced by the progress of the disease.

[All this is no doubt very ingenious, and may be of use in some cases to those who are commencing the study of auscultation. Knowing, however, the ever ready and inexhaustible stock of materials for auscultation in the *living* body that exist around every enquirer, we cannot but see in the investigations of M. Petrequin, rather the luxury or wantonness of riches than the resource of poverty: we are, nevertheless, far from wishing to depreciate his labours, and even recommend them to the notice of our students in the dissecting room.]

*Revue Médicale. Mars*, 1838.

---

*On the Use of the essential Oil of Turpentine in Diseases of the Eye.*
By Dr. A. Trinchinetti.

The author's experience induces him to place great confidence in the oil of turpentine in the slow and deep-seated inflammations of the eye, especially in those that do not yield to antiphlogistic measures. Cases are given proving its utility in chronic inflammation of the iris or ciliary bodies, and in incipient gangrene of the cornea, all of these following the operation for cataract; in the chronic stage of rheumatic iritis, or even in the outset, if it be mild; in traumatic iritis, ulcers of the cornea, onyx and incipient glaucoma. The oil should be administered in emulsion, the dose varying from half a drachm to four drachms daily.* The phenomena generally following its use are diminution or cessation of pain, a sense of general comfort, contraction of the vessels with gradual disappearance of the inflammatory fulness and lachrymation; the easy dispersion of the matter effused into the anterior chamber or between the lamellæ of the cornea. Occasionally a sensation of weight and burning in the stomach, especially after full doses, was felt, and in some rare cases was sufficiently troublesome to prevent the further administration of the drug. Instead of producing a purgative effect, it caused constipation; the urine became abundant, of violet odour, was passed without pain and deposited a reddish sediment.

*Giornale delle Scienze Med.-Chir.* No. 26. *Agosto*, 1836.

---

*On the Employment of Chlorine in Acute and Chronic Bronchitis.*
By Dr. Toulmouche, Physician to the Prison of Rennes.

Dr. T. has been in the habit, since 1831, of employing chlorine in the treatment of bronchitis: he has found its effects beneficial in acute as well as in chronic bronchitis, and sometimes in pituitous catarrh. The method of employing it is similar to that generally adopted. A dose, varying from ten to one hundred drops of solution of chlorine, is poured upon hot water in an inhaling vessel, through which the patient breathes: from thirty to forty drops is the dose generally employed. Bronchitis is endemic in the prison of Rennes, which is situated on low and damp ground, surrounded with water. In its acute form, it presents the ordinary symptoms, and requires antiphlogistic treatment. The inhaled chlorine has generally been well borne; and, if it occasionally appeared to irritate at first, its administration was resumed with success in a few days. Chronic bronchitis is particularly frequent in the prison, on account of the dampness of the prisoners' workshops, as well as the generally unhealthy situation. When not complicated with emphysema of the lungs, chronic bronchitis is much benefited by chlorine, which produces no irritation, but alters the character and diminishes the quantity of the bronchial discharge. In cases of pituitous catarrh, or bronchorrœa, where the sputa are generally transparent, but little benefit is derived from chlorine. In addition to the inhalation, a solution of chloruret of soda was occasionally given; but the patients were with difficulty induced to take it, on account of its very bad taste. In four years and a half, the chlorine treatment was employed in 309 cases,

* The best formula for its exhibition is that proposed by Mr. Carmichael in 1829.

—228 female and 81 male,—although the males were to the females in the prison as 150 to 180. Of 141 cases of acute bronchitis in female prisoners, 134 were cured after a treatment of from five to fifteen days. Of 65 cases of chronic bronchitis, 51 were cured after a treatment of from five to ninety-eight days: 4 died; the rest must be considered as not relieved. When chronic bronchitis was complicated with phthisis, no benefit was derived from inhalation. Of 65 cases of acute bronchitis in males, all were cured after treatment of two to ten days. Of 12 chronic cases, 10 were cured after using the chlorine from ten to forty days. Dr. Toulmouche cannot state precisely the duration of the disease in cases previously treated in the ordinary method; but it is much greater than in those treated with chlorine. *Gazette Médicale, June,* 1838.

---

*Of the Symptoms of the earliest Stage of Phthisis Pulmonalis.* By M. Fournet.

[The following observations are much less novel than the author seems to imagine; and the accuracy of some of them we, moreover, question: still as no point in practical medicine is more important than the one here sought to be elucidated, we give M. Fournet's remarks as we find them, and without comment.]

The endeavour of M. Fournet has been to detect pulmonary tubercles when few in number and in a state of crudity. His investigations have been carried on under the eye of M. Andral, whose testimony is added in support of his opinions.

*General Phenomena.* Instead of that state of happy illusion which accompanies phthisical patients, even when on the verge of the tomb, these patients, in the early stage, are always disturbed, morose, and constantly occupied with melancholy thoughts about themselves; and it is only when the disease has made some progress that the state of confidence is manifested.

*Antecedent History.* M. Fournet regards other circumstances besides hereditary disposition as exercising a strong influence on the development of phthisis; such as excess in spirituous liquors, extreme venery, grief, too hard bodily work, residence in a bad atmosphere, bad food: and he says that when the disease develops itself under the influence of these causes, its form is commonly miliary and its progress rapid; whilst the constitutional phthisis is chronic, and characterized by large tubercles.

*Local Signs.* In the first period of pulmonary phthisis, the soft and easy character of the inspiration and expiration is changed to one of hardness, harshness, with an impression as if the sound was produced with difficulty. The sounds become dry, instead of imparting, as in the normal state, a sensation intermediate to moisture and dryness. The inspiration is less in duration; sometimes is unchanged in intensity; at others, this is increased. The expiration increases successively, both in intensity and duration; and this augmentation is in direct relation with the more or less advanced state of the disease. The changes in the "key" (*timbre*) are subsequent to those in the duration and intensity of the respiratory murmurs, and to the harshness and dryness of them. They are always first heard in expiration, and afterwards during inspiration. Two sounds, described by M. Fournet as "bruit de froissement pulmonaire" and "bruit de craquement," belong equally to the first period of phthisis. There is a dry and a moist condition of the "bruit de craquement." The dry *craquement* commonly exists only during inspiration, and it extends also to the act of expiration in proportion as its character changes from dryness to moisture. The "bruit de froissement pulmonaire" is scarcely heard except during inspiration. M. Fournet has further established the fact that the sounds of the heart are louder in that clavicular region where the tubercular infiltration is the more advanced: and this fact is of considerable importance, if, as is stated by M. Fournet, the disease, even in its early period, is more advanced on the right side; since, in this case, the transmission of the sounds of the heart more forcibly beneath the right clavicle is contrary to the normal condition.

In percussion, M. Fournet thinks that as much is to be learned by touch as by auscultation. The vocal vibration is diminished in direct proportion to the degree of tubercular infiltration; excepting when a cavity is formed, which increases the vibration. M. Fournet has found, also, in very many cases of phthisis in their earliest period, that there is a sensible diminution of size of the summit of the chest; and, consequently, a flattening in front, and a sinking more or less considerable of the supra and sub-clavicular regions. He measures this degree of sinking of the summit of the chest by stretching a straight line between two fixed points, the middle of the clavicle and the nipple, and by measuring the radius of the arc of the circle which subtends this straight line. This mode of measurement is more exact than that which is practised above the moveable mass of the great pectoral and dorsal muscles. And, lastly, M. Fournet has analyzed the sensations experienced by patients in different parts of the chest, and he has ascertained the existence of a sensation of difficult, straitened, and incomplete respiration on that side where the tubercular infiltration was at its maximum: and this side becomes affected with a painful resonance when the patient speaks or coughs somewhat forcibly, or when percussion is practised comparatively upon the corresponding parts of the thorax. The voice, too, becomes feeble, is formed with effort, is veiled, somewhat hoarse, and of a deeper tone. Of these signs, some appear sooner than others. It is by a comparison of them, and of their relations and connexions one with another, the predominance of one over another, &c., that the diagnosis is formed: and on the above grounds the author, eight months since, diagnosticated the existence of infiltrated tubercles in the summit of the lungs of a strong and healthy young man, aged seventeen, who had never had hæmoptysis, was not born of tubercular parents, but had been admitted under the care of M. Andral for an eruptive fever. At the present time this patient has all the signs of cavities in the upper part of the chest.

*Bulletin de l'Academie Royale de Médecine.* No. 13. 1838.

